# Public hospital reforms in China: the perspective of hospital directors

**DOI:** 10.1186/s12913-019-3954-z

**Published:** 2019-02-28

**Authors:** Ju Huang, Tao Dai

**Affiliations:** The Institute of Medical Information, Chinese Academy of Medical Sciences and Peking Union Medical College (CAMS & PUMC), No.3 Yabao Road, Chaoyang District, Beijing, People’s Republic of China 100020

**Keywords:** Perspective, Health policy, Reform, Survey

## Abstract

**Background:**

To understand the attitudes and opinions of directors of large public hospitals in China on reform policies of public hospital.

**Method:**

A cross-sectional survey utilizing questionnaires of the Survey of Attitudes to Public Hospital Reform by Directors was conducted in 2014. The respondents were randomly selected in 100 tertiary public hospitals in 62 cities of 31 provinces in China by stratified multistage random cluster sampling method. 178 directors and associate directors working in tertiary public hospitals were involved. Standard descriptive statistics were used to describe and summarize the data.

**Results:**

The measure of increasing government subsidies ranked first in the list of concerns expressed by responders (*N* = 149, 83.7%); while implementing clinical pathways ranked lowest in the list of concerns (*N* = 34, 19.7%). More associate directors (*N* = 64, 70.3%) were concerned over the measures of removing drug mark-ups than directors (*N* = 45, 51.7%) (χ^2^ = 6.49, *p* = 0.01). In addition, 75.8% of responders were concerned over the policy of salary system reform, while only 14.5% of them were satisfied with their current income level. What’s more, more than half responders were concerned over the policy of adjusting pricing policies (*N* = 127, 71.4%) and removing drug markups (*N* = 109, 61.2%).

**Conclusion:**

In healthcare reform, the financial security for the hospitals should be considered as a priority by the policy-makers, without the reform goals cannot be achieved. Thus, an incentive mechanism needs to be established in China to guide the director to focus on the medical quality.

## Background

Healthcare reform is a challenging global issue. The public hospital reform is not only important but also difficult in China. The public hospitals consume a large proportion of health resources of both outpatient and inpatient care in China. In 2015, 88.0% accounted for the total number of outpatient visits in public hospitals, and 85.3% accounted for the total number of in-patients [1]. The funds for public hospitals gained primarily from the three health insurances, out-of-pocket payments, and drug mark-ups before 2017 [2–4]. Therefore, the public hospitals have been criticized for their profit-seeking behaviors [4]. On one side, the basic medical services and essential drugs are inadequately provided by public hospitals due to fees lower than the initial costs [5]. On the other side, the rapid expansion of the tertiary public hospitals came with the escalating expenditures of medical care [4].

Several countries have implemented healthcare reforms over recent years but have encountered a range of difficulties. However, the Chinese government has selected some urban and rural hospitals for pilot reforms since 2010. Some of these which were listed in the document of Planning and Implementation Plan of Deepening Health System Reform during “The Twelfth Five-year Plan” (here after referred as the Plan) are described here.

### Reform of payment mechanism

The drug mark-ups were eliminated, and the funds for public hospitals gained primarily from the three health insurances and out-of-pocket payments [4]. This indicated that the local government needs to arrange additional funding for the public hospitals’ financial survival [6, 7].

### Adjusting pricing policies

The prices of the professional services provided by the healthcare workers (HCWs) increases promote fees cover the initial costs, and the prices of high technologies for the diagnosis and treatment reduced to provide incentives to HCWs to provide cost-effective medical services.

### Reform of health insurance payments

To control the expenditures and improve the quality, in combination with the clinical pathways of the diseases, the reform is effectuated by promoting the payments based on DRGs and global budget.

### Establishing the modern hospital management system

This reform authorizes the public hospital to appropriate the income distribution that would increase the performance-related-pay to effectively motivate the HCWs, while the authority over capital is assigned to a special administrative institution (Hospital Authority), which would restrict the power of the directors with respect to the hospital investment to restrain infinite expansion of large public hospitals.

### Defining the power of government in public hospital administration

This reform aimed to intensify the government supervision on hospitals and control the extension of government administration, which could also motivate the health sectors.

Although the above policies of health reform have been initiated, the problem has not been resolved such that the tertiary public hospitals are rapidly expanded [4, 8]. One of the key reasons is that the central government and people support the reform; however, the public hospitals and HCWs, especially the tertiary public hospitals supported the reform verbally but actually were against the reform. This is one of the main reasons for the failure to achieve the goals of the reform.

In China, public hospitals are comprised of national hospitals, provincial hospitals, city hospitals, and district or county hospitals owned by the governments at a relevant level that dismiss and appoint their directors and empower them to manage hospitals. Thus, a de facto principal-agent relationship is formed between the government and the director [9]. The hospital directors are responsible for not only the business and development of public hospitals but also the goals of the government at appropriate level, while the associate director or other hospital administrators may only be concerned about the financial position or operation results of the hospitals. Their opinions and attitudes would directly affect the implementation of the reform measures in public hospitals. In some cases, the goals of public hospital principal (government) and the agent (director) are not identical, which might lead to the deviation of the behaviors of the hospitals from the reform goal [10]. However, rare literature is available on the survey of the views of large public hospital directors on reform due to the low response rate. Therefore, it is necessary to investigate the views of the directors of public hospitals, especially those of tertiary public hospitals, which account for a majority of the resources in healthcare service in China in order to understand their attitudes and opinions on reform-related policies.

The present study hypothesized that the public hospital directors are most concerned about the reform policies about financial position or operation results of the hospitals, and associate directors are concerned about the financial position or operation results of the hospitals than the directors. Herein, we not only identified the problems encountered by the public hospital reform and provided empirical support for promoting the public hospital reform in China and worldwide but also analyzed the health reform issues of international concern from the perspective of the public hospital directors.

## Methods

### Sampling of study population

The respondents were public hospital directors from tertiary public hospitals in China, including directors and associate directors who were involved in the decision making [11]. The Chinese Hospital Association (CHA) organized and carried out the survey. The respondents were randomly selected from 100 tertiary public hospitals in 62 cities of 31 provinces by stratified multistage random cluster sampling method. Then, 200 selected directors and associate directors were interviewed through face-to-face or telephone interview by CHA investigators between July and August of 2014. Among the 200 respondents selected, 178 returned the questionnaires, and the response rate was 89%, of which, 87 were directors and 91 were associate directors.

### Survey design

A cross-sectional survey utilizing questionnaires was conducted. Thus, we developed a survey instrument, the Survey of Attitudes to Hospital Reform by Directors (SAHRP). The SAHRP is based on the document of the Plan and previous Health Care Worker Satisfaction Questionnaires. The SAHRP consists of 4 main areas: (1) individual characteristics, (2) the most concerned policies for the directors in public hospital reform, (3) the views of the directors on key measures proposed in the document of the Plan, and (4) attitudes of the directors on the public hospital governance and income.

Firstly, the most concerned policies for the directors in public hospital reform were investigated using the following item: “Which of the following policies of public hospital reform are of great concern to you?” It is a multiple choice question, and the answers were eight specific measures in six aspects of the reform content included in the document of the Plan, including the following: (I) Intensifying the government support for public hospitals, and the specific measure is increasing subsidies. (II) Reform of financing mechanisms, including adjusting pricing policies of medical services and removing the drug mark-ups in public hospitals [6, 7]. (III) Controlling the rise of medical cost, especially reforming payment methods such as DRGs, capitation, and global budgets [12, 13]. (IV) Establishing the modern hospital management system, including the reform of personnel affairs and payroll distribution and clarifying the responsibility and power of public hospital directors. (V) Defining powers of government, such as intensifying the government supervision. (VI) Innovating hospital management and service, such as implementing clinical pathways.

Secondly, the directors’ views towards the key measures proposed in IPDHCR were explored using the following five questions: (1) How much percentage of the total income of a hospital should the government subsidies account for? (2) Which of the following financial support should be provided by the government for public hospitals? (3) How to fill in the financial gaps when drug markups are removed? (4) Which of the following payments in health insurance should the hospitals apply for? (5) What are the main factors affecting the participation of medical professionals in public hospital reform? These were all multiple choice questions except item (1), and responses are summarized in Table 3.

Finally, directors’ attitudes toward hospital governance and income were collected by the following three items: (6) A corporate governance structure should be established, such as the board of directors and management committee in public hospitals; (7) The overall income level of HCWs in the current hospital is reasonable; (8) I am satisfied with my current income level as compared to my contributions to hospital development. The responses were scored on a 5-point Likert scale ranging from “strongly agree” (5) to “strongly disagree” (1). Each scale was tested for internal consistency on the pilot sample, and modifications were made according to the feedback.

### Data analysis

The samples were divided into the director and the associate director groups. Standard descriptive statistics were used to describe and summarize the data. The categorical data were analyzed using Pearson’s chi-square or Fisher’s exact test as appropriate. The analysis of variance was used to compare the mean values in both groups. All data were analyzed using SPSS 18.0. A *p*-value < 0.05 was considered as statistically significant.

## Results

### Baseline information

Table 1 shows the responders’ characteristics. The average age of the respondents (*N* = 178) was 49.9 years. The directors (*N* = 87, 49%) were about 3 years older than the associate directors (*N* = 91, 51%), and the difference was statistically significant (*F* = 15.478, *p* < 0.0001). The respondents were predominantly male (*N* = 136, 76.4%). A total of 61.2% responders had a Bachelor degree, and the main profession of the responders was medicine, which accounted for 77.5%, while the management specialty accounted for only 12.9% of the individuals. However, no statistically significant differences were detected with respect to gender, educational level, and professional specialty between the directors and associate directors.

**Table 1:** Respondent characteristics

*Respondent*		Total *N* = 178	Director *N* = 87	Associate Director *N* = 91	*p*	X^2^
Gender	Male	136 (76.4)	65 (74.7)	71 (78.0)	0.603	0.270
	Female	42 (23.6)	22 (25.3)	20 (22.0)		
Education	Postgraduate	47 (26.4)	26 (29.9)	21 (23.1)	0.497	1.399
	Undergraduate	109 (61.2)	52 (59.8)	57 (62.6)		
	Junior college and below	22 (12.4)	9 (10.3)	13 (14.3)		
Professions	Medicine	138 (77.5)	62 (71.3)	76 (83.5)	0.086	4.913
	Management	23 (12.9)	16 (18.4)	7 (7.7)		
	Others	17 (9.6)	9 (10.3)	8 (8.8)		

Note: Numbers in parentheses are percentages of the sample shown exclusive of missing data, null hypothesis is “no difference”

### Reform measures which were of great concern to directors

Table 2 summarizes the information on the matters of concern for the responders with respect to each measure of the public hospital reform, their orders were as follows:

**Table 2:** Reform measures which were of great concern to directors

Policies	Specific Measures	Total *N* = 178		Director *N* = 87		Associate Director *N* = 91		
		Rank	No.(%)	Rank	No.(%)	Rank	No.(%)	*p* (X^2^)
(I)	Increasing subsidies	1	149 (83.7)	1	72 (82.8)	1	77 (84.6)	0.737(0.112)
(II)	Adjusting pricing policies	3	127 (71.4)	2	65 (74.7)	4	62 (68.1)	0.398(0.713)
	Removing drug mark-ups	4	109 (61.2)	5	45 (51.7)	3	64 (70.3)	0.011(6.486)
(III)	Reforming payment methods such as DRGs, capitation and global budgets	6	78 (43.8)	6	42 (48.3)	6	36 (39.6)	0.241(1.372)
(IV)	Reform of personnel affairs and payroll distribution	2	135 (75.8)	3	62 (71.3)	2	73 (80.2)	0.163(1.947)
	Clarifying power and responsibility of public hospital directors	5	103 (57.9)	4	58 (66.7)	5	45 (49.5)	0.020(5.407)
(V)	Intensifying the government supervision	7	59 (33.1)	7	24 (27.6)	7	35 (38.5)	0.123(2.374)
(VI)	Implementing clinical pathways	8	34 (19.7)	8	20 (21.8)	8	14 (17.6)	0.197(1.664)

Abbreviation: (I) Intensifying the government support for hospital

(II) Reform of financing mechanisms

(III) Controlling the rise of medical cost

(IV) Establishing the modern hospital management system

(V) Defining powers of government

(VI) Innovating hospital management and service

Numbers in parentheses are percentages of the sample shown exclusive of missing data, null hypothesis is “no difference”

Table 2 shows the numbers of responders who are concerned about each measure of the public hospital reform as follows: First, increasing subsidies (*N* = 149, 83.7%)*;* Second, reform of personnel affairs and payroll distribution (*N* = 135, 75.8%); Third, adjusting pricing policies (127, *N* = 71.4%); Fourth, remove drug mark-ups (*N* = 109, 61.2%); Fifth, clarifying the power and responsibility of hospital directors (*N* = 103, 57.9%); Sixth, reforming payment methods (*N* = 78, 43.8%); Seventh, intensifying the regulatory responsibilities of the government (*N* = 59, 33.1%); Eighth, implementing clinical pathways (*N* = 34, 19.7%). Therefore, the measure of increasing government subsidies ranked first in the list of concerns expressed by responders; while implementing clinical pathways ranked lowest in the list of concerns.

The numbers of directors and associate directors are also sorted in Table 2, which showed more associate directors were concerned over the measures of removing drug mark-ups than the directors (χ^2^ = 6.49, *p* = 0.01). A total of 66.7% of the directors was concerned over clarifying the power and responsibility of hospital directors*,* which was significantly higher than that of the associate directors (49.5%) (χ^2^ = 5.41, *p* = 0.02*).* However, no significant difference was observed in the number of responders on the remaining measures between the directors and associate directors. The results revealed that the directors might be more concerned about the power and responsibility of the hospital directors as compared to the associate directors, while the associate directors may be rather concerned about the hospitals’ financial position.

### Views of hospital directors on the key measures

Specifically, concerning the measure of increasing subsidies*,* the responders considered that the average government subsidies should account for 50.8% of the total hospital income (Table 3). In addition, more than a half of the responders (55.1%) considered hat the government should provide financial support to public hospitals by special subsidies*.* In terms of reform of financing mechanisms*,* more than 80% of the responders considered that two methods such as adjusting the medical service prices and increasing the government subsidies were efficient in filling the financial gaps when drug markups were removed. With respect to controlling the medical cost, 51.1% of the responders considered that the case-based payment should be applied in health insurance, while only 29.8% of the responders considered that the global budget should be applied in health insurance. The proportion of associate directors (22.0%) who supported global budget was lower than that of the directors (37.9%) *(*χ^2^ = *5.41, p = 0.02).* Finally, 82% of the respondents considered that no improvement in the income was the primary factor affecting the participation of medical professionals in public hospital reform.

**Table 3:** Views of the hospital directors on the key measures

	Total *N* = 178	Director *N* = 87	Associate Director *N* = 91	*p* (X^2^)
(1) How much percentage of total income of a hospital should the government subsidies account for^1^	50.8(23.0)	48.5(21.2)	53.4(24.7)	*F* = 1.139 *P* = 0.289
(2) Which of the following financial support should be provided by government for public hospitals				
*Providing subsidy on capitation*	87(48.9)	44(50.6)	43(47.3)	0.658(0.196)
*Providing subsidy on beds*	78(43.8)	30(34.5)	48(52.8)	0.014(6.027)
*Providing subsidy on performance*	69(38.8)	35(40.2)	34(37.4)	0.695(0.154)
*Special subsidies*	98(55.1)	51(58.6)	47(51.7)	0.350(0.874)
*Service purchase by government*	85(47.8)	47(54.0)	38(41.8)	0.102(2.682)
(3) How to fill the financial gaps when drug mark-ups are removed				
*Increasing government subsidies*	149(83.7)	70(80.5)	79(86.8)	0.251(1.316)
*Adjusting the medical service prices*	159(89.3)	78(89.7)	81(89.0)	0.889(0.019)
*Charging pharmaceutical service fee*	96(53.9)	51(58.6)	45(49.5)	0.220(1.505)
*Improving the efficiency of hospital running*	95(53.4)	45(51.7)	50(55.0)	0.667(0.185)
(4) Which of the following payments in health insurance should the hospitals apply for				
*Fee-for-service*	63(35.4)	27(31.0)	36(39.6)	0.234(1.414)
*DRGs*	91(51.1)	41(47.1)	50(55.0)	0.297(1.088)
*Global budget*	53(29.8)	33(37.9)	20(22.0)	0.020(5.414)
(5) What are the main factors affecting the participation of medical professionals in public hospital reform				
*No improvement in income*	146(82.0)	73(83.9)	73(80.2)	0.522(0.410)
*No improvement in social status*	132(74.2)	62(71.3)	70(76.9)	0.389(0.743)
*Conflict between doctors and patients*	128(71.9)	57(65.5)	71(78.0)	0.064(3.443)

Numbers in parentheses are percentages of the sample shown exclusive of missing data, null hypothesis is “no difference”, 1 Mean (Std),using analysis of variance

### Attitudes of hospital directors’ on hospital governance and income

The results of the attitudes are shown in Table 4. A majority of the respondents considered the need to establish a corporate governance structure including the board of directors and management committee in public hospitals, and the proportion of associate directors was 77.8%, which was higher than 58.1% of the directors (χ^2^ = 16.18, *p* = 0.003). Concerning income, only 25.3% of the respondents considered that the overall income level of HCWs in the current hospital was reasonable, and the proportion of associate director was only 18.2%, which was lower than 32.6% of the directors (χ^2^ = 14.67, *p* = 0.005). Only 14.5% of the respondents were satisfied with their current income level, and no significant difference was detected between the directors and associate directors.

**Table 4:** Attitudes of hospital directors’ on hospital governance and income

	Total *N* = 178	Director *N* = 87	Associate Director *N* = 91	*p* (X^2^)
	Agree	Agree	Agree	
(6) A corporate governance structure should be established, such as the board of directors and management committee in public hospitals	120(67.4)	50(57.5)	70(76.9)	0.003(16.182)
(7) The overall income level of HCWs in the current hospital is reasonable	44(25.3)	28(32.6)	16(18.2)	0.005(14.671)
(8) I’m satisfied with my current income level as compared to my contributions to hospital development	25(14.5)	12(14.1)	13(14.8)	0.528(3.181)

Abbreviation: *HCW* health care worker

Numbers in parentheses are percentages of the sample shown exclusive of missing data, null hypothesis is “no difference”

## Discussion

The results of the present study revealed that the reform related to the hospital financial position ranked first in the list of concerns expressed by directors, the reform on the hospital salary system ranked second, and the reform on power and responsibility of public hospital directors ranked third, while implementing clinical pathways ranked lowest in the list of concerns by both directors and associate directors*.* These findings confirmed the hypothesis that public hospital directors were maximally concerned about the reform policies on financial position or operation results of the hospitals, and associate directors were more concerned about the financial position or operation results of the hospitals than directors.

The reform on the hospital salary system ranked second in the list of concerns expressed by the directors, and most of them were dissatisfied with their incomes. The compensation system reform of public hospitals in China is yet lagging [14]. Furthermore, the income of HCWs is directly linked to the department income in most public hospitals, which effectively motivates the HCWs, but leads to profit-seeking behavior of public hospitals, such as over prescription and examination among HCWs. On the other hand, the cap on salaries at public hospitals restricts the reasonable growth in income of HCWs, thereby resulting in the dissatisfaction with their income [15–17]. The results showed that only 25.3% of the hospital directors considered that the overall income level of HCWs in the current hospital was reasonable, and only 14.5% of the hospital directors were satisfied with their current income level.

The reform on the power and responsibility of the public hospital directors ranked third. The investigation showed that 67.4% of the hospital directors considered that the public hospitals needed to establish a corporate governance structure such as the board of directors and management committee. In the institutional arrangements of powers and responsibilities between the government and public hospital directors, although the regulations and regulatory documents extend some rights to the directors, major decision-making powers of the financial budget and final account of the hospital have not yet been defined clearly [10]. The corporate governance structure reform in public hospitals will help to clarify the division of power and responsibilities between the directors and the government [18].

Interestingly, directors were mainly concerned about the reform policies on financial position or operation results of the hospitals, including increasing subsidies and adjusting pricing policies, rather than implementation of clinical pathways. Strikingly, due to irrational medical service price system and insufficient subsidies*,* many public hospitals had encountered financial problems for several years. Thus, improving the medical quality might increase the hospital operating costs, and eliminating drug mark-ups will reduce the hospital income [6], which would increase the hospitals’ financial burden. Therefore, the reform policy that implementation of clinical pathways or eliminating drug mark-ups are inconsistent with the director’s goals will achieve half the result. For example, since 2017, all public hospitals have eliminated drug mark-ups in succession. However, due to insufficient subsidies and motives for cost-efficiency, hospital expenditures for the diagnosis and treatment continue to rise, also causing a continual increase in the medical expenses per hospital admission [19].

What’s more, the reform of public hospitals in China has been implemented for approximately 9 years; however, the result showed that the excessive rise of medical expenses was not contained, while on the other hand, most public hospital directors and HCWs were still not satisfied with their income. The main problem is that the remuneration of HCWs in public hospitals in China primarily depends on the number of medical services they provide to patients, rather than the medical outcomes. Conversely, in the developed countries, the remuneration of HCWs in public hospital does not depend on the number of medical services they provided [20], and hence, the leading issue of health reform is the improvement of health service efficiency. Therefore, in the developed countries, the implementation of clinical pathways that aim to link the practice and optimization of clinical outcomes while maximizing the clinical efficiency is a critical measure for health reform [21]. However, it is not valued by public hospital directors in China. It suggested that without the rational payment mechanisms (including medical service price system) and a long-term government financing mechanism, the directors’ interests would focus on the financial position of the public hospitals and salaries of HCWs, but not on the improvement of medical quality and management. Therefore, in the healthcare reform, the financial security for hospitals should be prioritized while implementing the reform measures; otherwise, the reform might not achieve the intended goals. Secondly, improving the quality of medical services should be of the concern for all countries in their health reforms. Moreover, in China, an incentive mechanism should be established to guide the directors to implement the clinical pathways in public hospitals.

### Limitations

The present investigation was initiated in 2014, and thus, the collected data does not reflect the latest status. However, the issues in the reform of public hospitals in China from the perspective of a hospital director are reflected by the data. Also, the specific innovative results can provide a basis for the international health reform. Secondly, the directors of the tertiary general hospitals were mainly investigated in this study, while those of the second-class hospitals were not investigated. Therefore, the study results mainly represent the opinions of the directors from the large-scale general hospitals on reform.

## Conclusions

Without the rational payment mechanisms and a long-term government financing mechanism, the public hospital directors’ interests would focus on the financial position of the hospitals and salaries of HCWs but not on the improvement of the medical quality and management. In healthcare reform, the financial security for the hospitals should be considered as a priority by the policy-makers, without the reform goals cannot be achieved. Thus, an incentive mechanism needs to be established in China to guide the director to focus on the medical quality.

## Abbreviations

CHA: Chinese Hospital Association
DRGs: Diagnosis-related groups
HCW: Health Care workers
SAHRP: The Survey of Attitudes to Hospital Reform by Directors
